# Plant Extracts as Modulators of the Wound Healing Process—Preliminary Study

**DOI:** 10.3390/ijms26157490

**Published:** 2025-08-02

**Authors:** Anna Herman, Aleksandra Leska, Patrycja Wińska, Andrzej Przemysław Herman

**Affiliations:** 1 Chair of Drug and Cosmetics Biotechnology, Faculty of Chemistry, Warsaw University of Technology, Koszykowa 75 Street, 00-662 Warsaw, Poland; aleksandra.leska@pw.edu.pl (A.L.); patrycja.winska@pw.edu.pl (P.W.); 2 Department of Genetic Engineering, The Kielanowski Institute of Animal Physiology and Nutrition, Polish Academy of Sciences, Instytucka 3 Street, 05-110 Jabłonna, Poland; a.herman@ifzz.pl

**Keywords:** plant extract, wound healing, *Vitis vinifera*, *Cynara cardunculus*, *Rhodiola rosea*, *Rosa canina*

## Abstract

The treatment of chronic wounds is one of the most complex therapeutic problems of modern medicine. It leads to patients’ protracted recovery, generating high treatment costs. Herbal products may be useful in the treatment of chronic wounds via a wide range of pharmacological properties and multidirectional effects on the wound healing phases. The study aims to determine the ability of selected plant extracts to modulate the processes involved in wound healing. The antimicrobial (MIC, MBC, MFC) and antioxidant (ABTS, DPPH) activities, cytotoxicity (MTT test), scratch wound test, and collagen assay were tested. *R. canina* (MBC 0.39 mg/mL) and *V. venifera* (MBC 3.13 mg/mL) extracts had bactericidal activities against *P. aeruginosa* and *S. aureus*, respectively. The *V. vinifera* extract showed the highest antioxidant activity in both ABTS (EC50 0.078 mg/mL) and DPPH (EC50 0.005 mg/mL) methods. The percentage of wound closure observed for *C. cardunculus*, *R. rosea*, and *R. canina* extracts with HaCaT, and *V. vinifera* extract with Hs27 cells was set as 100%. *V. vinifera* extract (50 μg/mL) stimulated collagen synthesis 5.16 times more strongly than ascorbic acid. Our preliminary study showed that some plant extracts may be promising modulators of the wound healing process, although further in-depth studies are necessary to determine their effectiveness in the in vivo model.

## 1. Introduction

The treatment of chronic wounds is one of the most complex therapeutic problems of modern medicine and leads to protracted recovery of patients, generates high costs of their treatment, and the need for everyday support in social life. Burn wounds, chronic wounds, and diabetic foot ulcers are examples of complicated wounds in which, despite appropriate treatment, the healing process is excessively extended in time [1,2,3]. These wounds are accompanied by chronic inflammation resulting from the inhibition of humoral and cellular immune response, which, in turn, increases the susceptibility of the wound to bacterial infections, which further delay and hinder the process of proper wound healing [4]. Moreover, chronic wounds are often accompanied by local wound hypoxia related to circulatory disorders, angiopathy, and neuropathy [5]. The occurrence of several disorders simultaneously makes the treatment of chronic wounds a significant therapeutic challenge. General guidelines for the treatment of chronic wounds include maintaining wound hygiene by cleaning it and protecting it from superinfection, regularly changing dressings, and supporting the process of epidermis regeneration [6]. The ointments and dressings available on the medical market contain compounds that maintain wound moisture or prevent bacterial infection but do not stimulate skin regeneration processes. Properly selected active compounds should influence the biochemical processes occurring in each of the four phases of wound healing, including the wound cleansing phase, the inflammatory phase, the proliferative phase, and the wound maturation phase [7]. Active compounds with multidirectional effects on the wound healing phases and simultaneously having antimicrobial properties may be useful in the treatment of chronic wounds.

Herbal products have an entrenched use in many branches of pharmacy and medicine as active ingredients of drugs. Plant-derived products are a broad and very diverse group in terms of chemical structure, often a mixture of several hundred different compounds, which explains their wide range of pharmacological effects. It was shown that *Vitis vinifera* (grape) seed extract is a rich source of polyphenols (70–80%), including catechins, epicatechin, gallic acid, quercetin, as well as procyanidins and proanthocyanidins known for their ability to scavenge free radicals, antimicrobial and anti-inflammatory activities [8,9,10,11]. *Cynara cardunculus* (artichoke) leaf extract and *Passiflora incarnata* extract are known to be rich sources of phenolic compounds [12,13]. *Pinus sylvestris* (pine) bark extract contains phenolic compounds like myricetin, eleutheroside, quercetin, proanthocyanidins, and volatile compounds (mainly α-pinene and β-pinene) responsible for antioxidant and antimicrobial activity [14,15]. *Syzygium aromaticum* flower buds extract is a rich source of eugenol (54.63%), eugenol acetate (21.57%), and caryophyllene (16.71%) with antimicrobial activity [16,17]. *Rhodiola rosea* root extract produces several bioactive compounds including salidroside, tyrosol, rosarin, rosavin, and rosin considered potentially beneficial in the treatment of chronic diseases [18,19]. *Scutellaria baicalensis* root extract poses baicalin, wogonin, and oroxylin A, known for their broad spectrum of biological effects, including antioxidant and anti-inflammatory [20]. *Smilax officinalis* root extract is a rich source of steroidal saponins with well-known anti-fungal and anti-inflammatory activities [21]. *Rosa canina* (rosehip) extract revealed a high content of bioactive compounds, primarily ascorbic acid and flavonols with antioxidant activity [22]. Boswellic acids, the main constituent of *Boswellia serrata* resin extract, affect the immune system via down-regulation of TNF-α and decrease in IL-1, IL-2, IL-4, IL-6, and IFN-γ [23]. Moreover, recent reports suggest that some herbal products and their active compounds stimulate the healing of postoperative wounds of various etiologies [24], burn wounds [25], and diabetic wounds [26]. It should be emphasized that a major advantage of plant products is their multidirectional action, as they often simultaneously affect several physiological processes occurring during individual phases of wound healing. Plant raw materials have many valuable biological activities, including antimicrobial, antioxidant, and anti-inflammatory properties. Furthermore, plant raw materials stimulate angiogenesis, proliferation, and migration of keratinocytes and fibroblasts, and activate growth factors, ultimately influencing the biochemical mechanisms of wound healing [24,25,26].

The study aims to determine the ability of selected plant extracts to modulate the processes involved in wound healing.

## 2. Results

### 2.1. Antimicrobial Activity of Plant Extracts

The MIC, MBC, and MFC of the plant extracts are shown in Table 1. The extracts from *R. canina* (MBC = 0.39 mg/mL) and *S. aromaticum* (MBC = 0.78 mg/mL) have the strongest bactericidal activities against *P. aeruginosa*. The extract from *S. aromaticum* (MBC = 0.78 mg/mL), *V. vinifera*, and *B. serrata* (MBC = 3.13 mg/mL) showed the strongest bactericidal activities against *S. aureus*. None of the plant extracts inhibited the growth of *C. albicans* in the concentration range tested.

### 2.2. Antioxidant Activity of Plant Extracts

**Figure 1:**
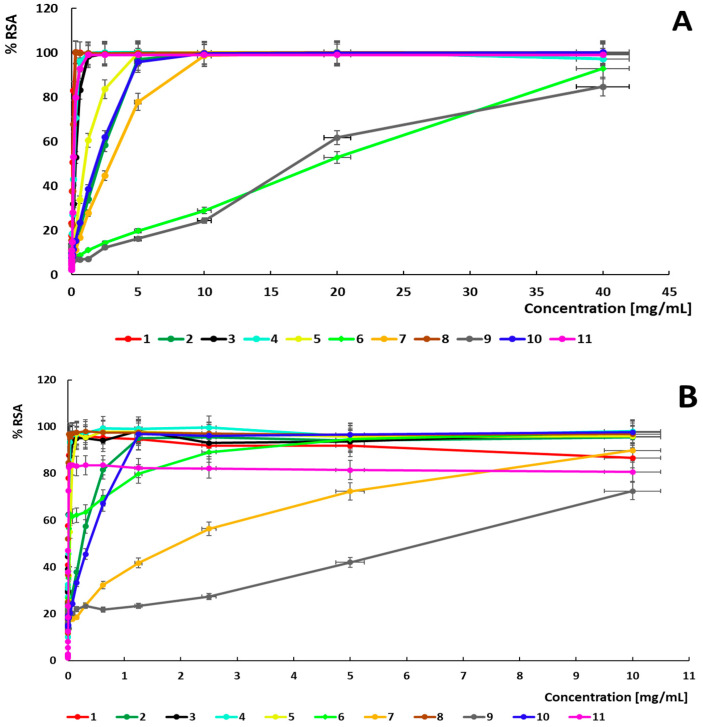
Radical scavenging activity (RSA) of plant extracts in ABTS (**A**) and DPPH (**B**) methods [%]. Legend: 1—*V. vinifera*; 2—*C. cardunculus*; 3—*P. sylvestris*; 4—*S. aromaticum*; 5—*R. rosea*; 6—*S. baicalensis*; 7—*S. officinalis*; 8—*R. canina*; 9—*B. serrata*; 10—*P. incarnata*; 11—Trolox.

The antioxidant activity of the plant extract based on ABTS and DPPH methods is shown in Figure 1. The *V. vinifera* extract has the most effective concentration of antioxidant compounds that results in 50% inhibition of radical formation (EC50 = 0.078 mg/mL; TEAC = 2.216; mg TE/g = 2437), even higher than those observed for Trolox (EC50 = 0.173 mg/mL, TEAC = 1.000) in the ABTS method. Extracts of *R. canina* (EC50 = 0.121 mg/mL; TEAC = 1.425; mg TE/g = 1362), *S. aromaticum* (EC50 = 0.264 mg/mL; TEAC = 0.654; mg TE/g = 874.8), and *P. sylvestris* (EC50 = 0.309 mg/mL; TEAC = 0.559; mg TE/g = 535.4) showed antioxidant activity comparable to Trolox. The antioxidant activity of *S. baicalensis* (EC50 = 19.602 mg/mL; TEAC = 0.009; mg TE/g = 7.895) was the weakest antioxidant activity observed in the ABTS method.

EC50 estimation, TEAC and TE of the antioxidant activity of plant extracts is shown in Table 2 for ABTS and DPPH methods. The *V. vinifera* (EC50 = 0.005 mg/mL; TEAC = 1.234; mg TE/g = 1538), *R. canina* extract (EC50 = 0.006 mg/mL; TEAC = 1.018; mg TE/g = 1052), and *P. sylvestris* (EC50 = 0.008 mg/mL; TEAC = 0.739; mg TE/g = 848.5) showed a similar effective concentration of antioxidant compounds to Trolox (EC50 = 0.006 mg/mL; TEAC = 1.000) in the DPPH method. The antioxidant activity of *B. serrata* (EC50 = 6.092 mg/mL; TEAC = 0.001; mg TE/g = 0.965) showed the weakest antioxidant activity observed in the DPPH method.

### 2.3. Effect of Plant Extracts on Viability of HaCaT and Hs27 Cell Lines

**Figure 2:**
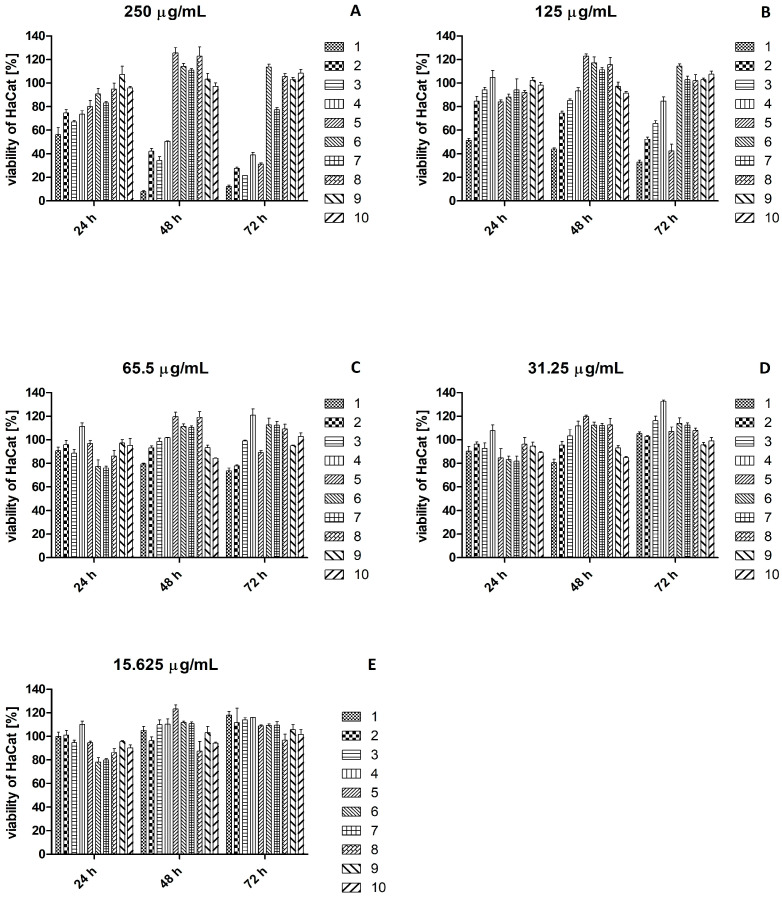
Viability of HaCaT cells after the treatment with plant extracts at a concentration 250 μg/mL (**A**), 125 μg/mL (**B**), 62.5 μg/mL (**C**), 31.25 μg/mL (**D**), and 15.625 μg/mL (**E**). Legend: 1—*V. vinifera*; 2—*C. cardunculus*; 3—*P. sylvestris*; 4—*S. aromaticum*; 5—*R. rosea*; 6—*S. baicalensis*; 7—*S. officinalis*; 8—*R. canina*; 9—*B. serrata*; 10—*P. incarnata*.

**Figure 3:**
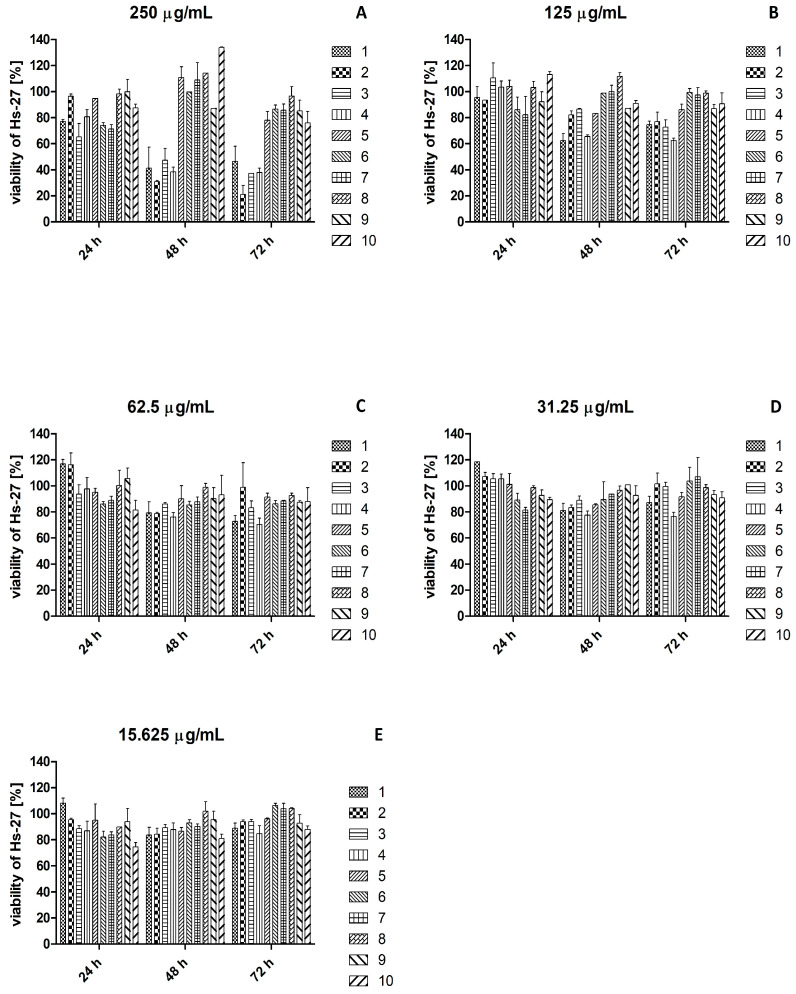
Viability of the Hs27 cell line after the treatment with plant extracts at a concentration 250 μg/mL (**A**), 125 μg/mL (**B**), 62.5 μg/mL (**C**), 31.25 μg/mL (**D**), and 15.625 μg/mL (**E**). Legend: 1—*V. vinifera*; 2—*C. cardunculus*; 3—*P. sylvestris*; 4—*S. aromaticum*; 5—*R. rosea*; 6—*S. baicalensis*; 7—*S. officinalis*; 8—*R. canina*; 9—*B. serrata*; 10—*P. incarnata*).

The effect of plant extracts on the viability of HaCaT cells (Figure 2) and Hs27 cells (Figure 3) was tested. All plant extracts used in concentrations below 62.5 μg/mL after 24 h, 48 h, and 72 h of incubation were safe for HaCaT cells. All plant extracts used at a concentration of 62.5 μg/mL after 24 h incubation were safe for Hs27 cells, while after 48 h incubation, cell viability was below 80% for *S. aromaticum* extract, and after 72 h for *V. vinifera* and *S. aromaticum* extracts. All plant extracts below the concentration of 31.25 μg/mL after 24 h, 48 h, and 72 h of incubation were safe for Hs27 cells.

### 2.4. Scratch Wound Healing Assay

**Figure 4:**
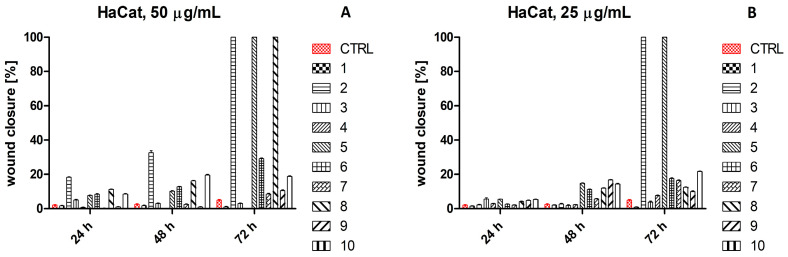
Wound closure [%] of the untreated human keratinocytes (HaCaT) and treated with 50 μg/mL (**A**) and 25 μg/mL (**B**) plant extracts after 24 h, 48 h, and 72 h incubation. Legend: 1—*V. vinifera*; 2—*C. cardunculus*; 3—*P. sylvestris*; 4—*S. aromaticum*; 5—*R. rosea*; 6—*S. baicalensis*; 7—*S. officinalis*; 8—*R. canina*; 9—*B. serrata*; 10—*P. incarnata*. A transparent bar chart refers to a wound closure percentage equal to 0.

*In vitro* scratch assay results for HaCaT and Hs27 cells are shown in Figures S1 and S2, respectively. The percentage of wound closure observed for 50 μg/mL of *C. cardunculus*, *R. rosea*, and *R. canina* extracts, as well as 25 μg/mL of *C. cardunculus* and *R. rosea* extracts after 72 h incubation with HaCaT cells, was set as 100% (Figure 4).

**Figure 5:**
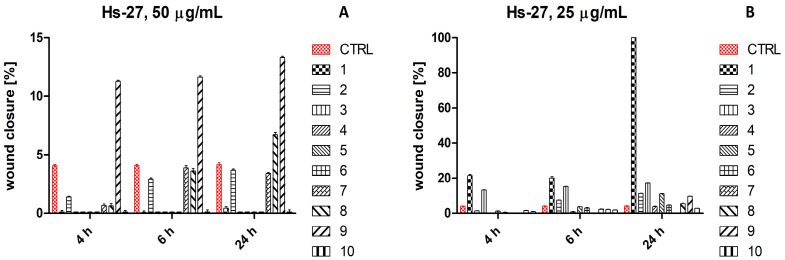
Wound closure [%] of the untreated human fibroblast (Hs27) and treated with 50 μg/mL (**A**) and 25 μg/mL (**B**) plant extracts after 4 h, 6 h, and 24 h incubation. Legend: 1—*V. vinifera*; 2—*C. cardunculus*; 3—*P. sylvestris*; 4—*S. aromaticum*; 5—*R. rosea*; 6—*S. baicalensis*; 7—*S. officinalis*; 8—*R. canina*; 9—*B. serrata*; 10—*P. incarnata*. A transparent bar chart refers to a wound closure percentage equal to 0.

In turn, the percentage of wound closure for 25 μg/mL of *V. vinifera* extract after 24 h incubation with Hs27 cells was set as 100% (Figure 5).

### 2.5. Collagen Assay

**Figure 6:**
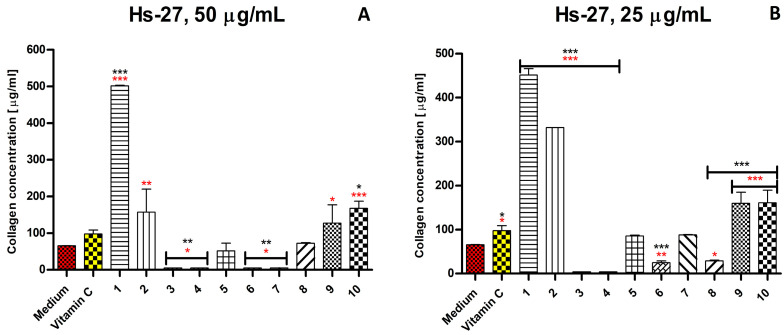
Collagen concentration [%] of the untreated human fibroblast (Hs27) and treated with 50 μg/mL (**A**) and 25 μg/mL (**B**) plant extracts. Statistical significance relative to the control was marked with red asterisks, while statistical significance relative to ascorbic acid was marked with black asterisks. The statistical significance of the differences was indicated in the figures with asterisks as follows: * *p* ≤ 0.05, ** *p* ≤ 0.01, and *** *p* ≤ 0.001. Legend: 1—*V. vinifera*; 2—*C. cardunculus*; 3—*P. sylvestris*; 4—*S. aromaticum*; 5—*R. rosea*; 6—*S. baicalensis*; 7—*S. officinalis*; 8—*R. canina*; 9—*B. serrata*; 10—*P. incarnata*.

*V. vinifera* extract in concentrations of 25 μg/mL (*p* ≤ 0.001) and 50 μg/mL (*p* ≤ 0.001) stimulates collagen synthesis 4.63 and 5.16 times more strongly than ascorbic acid, respectively (Figure 6). *C. cardunculus* extract in concentrations of 25 μg/mL (*p* ≤ 0.001) and 50 μg/mL (*p* ≤ 0.01) stimulates collagen synthesis 3.41 and 1,61 times more strongly than ascorbic acid. *B. serrata* (*p* ≤ 0.05 and *p* ≤ 0.001) and *p. incarnata* (*p* ≤ 0.001 and *p* ≤ 0.001) in both concentrations stimulate collagen synthesis 1.31–1.72 times stronger than ascorbic acid. *P. sylvestris*, *S. aromaticum, S. baicalensis,* and *S. officinalis* extracts at a concentration of 50 μg/mL, as well as *P. sylvestris, S. aromaticum, S. baicalensis*, and *R. canina* at a concentration of 25 μg/mL, inhibited the synthesis of collagen.

## 3. Discussion

Herbal products have many valuable biological activities, ultimately influencing the biochemical mechanisms of wound healing. Among all tested herbal extracts, *R. canina*, *V. venifera*, *R. rosea*, and *C. cardunculus* have biological properties that can be valuable in wound healing processes.

*V. vinifera* extract showed antioxidant and antibacterial activity, stimulates proliferation and migration of HaCaT and Hs27 cells, and collagen synthesis by fibroblasts. *V. vinifera* seed ethanol extract showed higher antioxidant activity than water extracts, and this property may be largely due to the activity of one of the main components of grape extract—proanthocyanidins [9,11]. Our results showed that *V. vinifera* seed extract inhibits the growth of bacteria (*P. aeruginosa, S. aureus*) but has no effect on yeast growth (*C. albicans*). It was previously reported that grape seed extract exhibited antibacterial activity against *Streptococcus mutants* [27], *Leuconostoc* sp. and *Micrococcus* sp. [28], *Bacillus cereus*, *B. coagulans*, *B. subtillis*, *S. aureus*, *Escherichia coli* and *P. aeruginosa* [29,30], but it did not exhibit a fungicide effect on *C. albicans* growth [31]. Several studies found that grape seed extract had anti-fungal activity against *C. albicans*, *Malassezia furfur*, and *Trichophyton mentagrophytes* [27,32]. The flavan-3-ols isolated from grape seed extract were recognized as the main anti-fungal agent against *C. albicans* [32]. *V. vinifera* extract does not show a cytotoxicity effect against HaCaT and Hs27 cell lines. The MTT assay demonstrated that grape seeds and skin extracts from *V. vinifera* had no cytotoxicity effect on HaCaT cells [33]. Some studies showed that *V. vinifera* extract stimulates wound healing [34,35,36,37,38,39]. The grape-skin powder increased hydroxyproline content, wound contraction, and decreased epithelialization time in the treated rats [34]. *V. vinifera* seed extract administered topically has a good potential to promote the repair of full-thickness wounds in rabbits [35], and skin wounds infected by *S. aureus* in diabetic rat models [36]. Furthermore, it demonstrates antibacterial efficiency against *S. aureus* in dermal application [37]. *V. vinifera* seed extract enhanced wound closure rates in rabbits, elevated TGF-β and VEGF levels, and significantly downregulated TNF-α and IL-1β levels [38]. Moreover, cream with *Aloe vera* and *V. vinifera* extract combination exhibits significant wound healing activity via increased VEGF and TGFβ1 in burn injuries [39]. Proanthocyanidins, a component of grape seed extract nanodispersion ointment, act via mobilization of the fibroblasts in the wound site and inhibit the inflammatory response through decreased expression of monocytes [40].

*R. canina* rosehip extract showed bactericidal activity against *P. aeruginosa*, strong antioxidant activity, stimulates migration of HaCaT, and collagen synthesis by Hs27 cells. It was demonstrated that rosehip flower ethanolic extract had the best antibacterial activity against *P. aeruginosa* and *E. coli* [41]. Among all hydrosoluble phytochemicals contained in rosehips, a high amount of ascorbic acid could be responsible for their biological activity [42]. A high concentration of ascorbic acid in rosehips is responsible for the strong antioxidant properties of *R. canina* extract and stimulation of collagen synthesis by fibroblasts. Ascorbic acid is involved in the hydroxylation of proline and lysine, which co-create collagen fibers and determine the stabilization of the protein structure of collagen [43]. The presence of ascorbic acid in *R. canina* extract accelerates re-epithelialization in the in vitro scratch assay [44]. It is well known that ascorbic acid contributes to wound healing through multiple mechanisms, including the regulation of inflammatory cytokine production, collagen synthesis, skin cell migration, wound closure, and re-epithelialization [45]. Furthermore, *R. canina* extract encapsulated into glycethosomes and allanthosomes may be potentially an efficient and innovative wound care product for accelerating skin wounds [46].

*R. rosea* extract stimulates the proliferation and migration of HaCaT cells, increasing collagen synthesis in Hs27 cells. *R. rosea* ethanolic extract possesses higher antioxidant activity compared to the aqueous extract or powdered raw material [47]. The bactericidal effect of aqueous *R. rosea* extracts was observed for *S. epidermidis*, *S. aureus*, *B. cereus*, *B. subtilis*, *L. monocytogenes*, *K. pneumoniae*, *Y. enterocolitica*, *P. mirabilis*, *P. aeruginosa*, and *E. coli* [48]. The oral daily administration (0.4 mg) of aqueous extract of *R. rosea* decreases *P. aeruginosa* infection after 7 days of treatment in mice [48]. The silver complex nanoparticles with *Resina draconis* extract and *R. rosea* extract loaded onto polyurethane nanofibers reached about 87.92% wound closure in diabetic mice, enhanced the migration and proliferation of fibroblasts, reduced the increased thickness of regenerated epidermis caused by diabetes, and the high expression and high lipid peroxidation levels of IL-1 b, IL-6, TNF a, iNOS, and MMP-9, and raise the low expression of VEGF [49].

*C. cardunculus* extract (artichoke) showed anti-staphylococcal and antioxidant activity, stimulates proliferation and migration of HaCaT, and increases collagen synthesis in Hs27. Petropoulos et al. [50] showed antioxidant activity dependent on different genotypes of *C. cardunculus* in DPPH scavenging activity. *C. cardunculus* seed extract showed antibacterial activity against *Bacillus aureus*, *Listeria monocytogenes*, *Enterobacter cloacae*, *Escherichia coli*, *Salmonella tympimurium*, and anti-fungal activity against *Aspergillus fumigatus*, *A. ochraceus*, *A. niger*, *Penicillium funiculosum*, *P. verrucosum*, *P. ochrochloron* [50]. Moreover, *C. cardunculus* extract significant effects against bacteria, such as *S. aureus*, methicillin-resistant *S. aureus*, *Bacillus cereus*, *B. subtilis*, *P. aeruginosa*, *Enterococcus faecalis*, *E. coli*, and fungi, such as *Aspergillus niger*, *Penicillium oxalicum*, *Mucor mucedo*, *Cladosporium cucumerinum*, and *C. albicans* [51]. The MDA-MB-231 cells migration assay showed that cells incubated with 35 μg/mL of *C. cardunculus* extract showed almost 100% wound closure after 24 h incubation [52]. Local prolonged treatment with 2% artichoke extracts (*Cynara scolymus*) improved collagen metabolism and attenuated the progression of inflammation in the skin aging model in rats [53]. Some research found that chitosan-based films loaded with ethanolic extracts from *Cynara cardunculus* leaves may be used as chronic wound dressings with anti-inflammatory activity [54].

*R. canina*, *V. venifera*, *R. rosea*, and *C. cardunculus* extracts possess multidirectional action, including antibacterial and antioxidant activity, stimulate proliferation and migration of keratinocytes and fibroblast cells, enhance collagen synthesis, and are not cytotoxic to skin cells. The applied methods allow for a preliminary assessment of whether selected plant extracts may be a potential stimulator of the wound healing process. In the conducted studies, out of ten plant extracts tested, only four exhibited promising biological properties valuable due to their influence on wound healing. However, further in-depth studies are necessary to determine their effectiveness in the in vivo model.

## 4. Materials and Methods

### 4.1. Plant Extracts

*Vitis vinifera* (grape) seed extract (standardized to 95% oligomeric proanthocyanidins), *Cynara cardunculus* (artichoke) leaf extract (standardized to 5% cynarin), *Pinus sylvestris* (pine) bark extract (standardized to 95% oligomeric proanthocyanidins), *Syzygium aromaticum* (sweet cap) flower buds extract (standardized to 25% polyphenols), *Rhodiola rosea* root extract (standardized to 3% rosavins and 3% salidrosides), *Scutellaria baicalensis* (Baikal skullcap) root extract (standardized to 85% baicalin), *Smilax officinalis* (Sarsaparilla) (gooseberry) root extract (standardized to 10% polyphenols), *Rosa canina* (rosehip) extract (standardized to 70% vitamin C), *Boswellia serrata* (frankincense) resin extract (standardized to 65% boswellic acids), *Passiflora incarnate* (passionflower) extract (standardized to 3.5% flavonoids) purchased in Medverita Group (Krakow, Poland). Lyophilized extracts were prepared in MilliQ water at a concentration of 100 mg/mL, left for 1 h, then centrifuged at 16,000 RCF/10 min, and subsequently, they were filtered with previously used sterile filters (0.22 μM).

### 4.2. Microorganisms

*Pseudomonas aeruginosa* ATCC 9027, *Staphylococcus aureus* ATCC 6538, and *Candida albicans* ATCC 10231 were used. The microorganisms were activated through double passaging: bacteria on TSA medium (Trypticase Soy Agar; BioMerieux, Marcy-l’Étoile, France) (37 °C, 24 h), and yeast on SDA medium (Sabouraud Dextrose Agar; BioMerieux, France) (30 °C, 48 h).

### 4.3. Cell Cultures

HaCaT (immortalized human keratinocytes) and Hs27 (human skin fibroblast cells) were purchased from the American Type Culture Collection (ATCC, Manassas, VA, USA). HaCaT and Hs27 cells were cultured in DMEM (Sigma-Aldrich Chemical Company, St. Louis, MO, USA) supplemented with 10% fetal bovine serum (FBS, Sigma-Aldrich Chemical Company, St. Louis, MO, USA), and antibiotics (100 U/mL penicillin, 100 μg/mL streptomycin) (Sigma-Aldrich Chemical Company, St. Louis, MO, USA). Cells were grown in 75 cm^2^ cell culture flasks (Sarstedt, Nümbrecht, Germany) in a humidified atmosphere of CO_2_/air (5%/95%) at 37 °C.

### 4.4. Determination of Minimum Inhibition Concentration (MIC), Minimum Bactericidal Concentration (MBC), and Minimum Fungicidal Concentration (MFC) of Plant Extracts by Broth Microdilution Methods

The MIC, MBC, and MFC of the lyophilized plant extracts were evaluated using the broth microdilution methods described by EUCAST [55,56]. Several colonies of overnight cultures for bacteria, and 48 h for yeast, were suspended in saline to obtain a density equal to 0.5 McFarland turbidity standard and then suspended in medium broth (Muller-Hinton for bacteria, Sabouraud for fungi) to 10^5^ CFU/mL. Lyophilised plant extracts were resuspended in medium broth in a concentration range of 0.78–100 mg/mL by twofold dilution in a 96-well microplate. Novobiocin (Oxoid, Basingstoke, Hampshire, UK) and cycloheximide (Oxoid, Basingstoke, Hampshire, UK) were positive controls. The sterility control (only medium broth) and the microorganisms growth control (bacteria/yeast in appropriate medium broth) were also prepared. All plates were incubated at 37 °C for 24 h. The MIC is the lowest concentration of the antimicrobial agent that inhibits the growth of the organism as detected by the unaided eye. For all the wells with clear broth, 100 μL of the sample was spread over the TSA and SDA agar plates. All bacterial plates were incubated at 37 °C for 24 h and yeast plates at 30 °C for 48 h, respectively. The MBC and MFC demonstrate the lowest concentration of the antimicrobial agent that results in bactericidal/fungicidal activity (no growth observed on plates).

### 4.5. Determination of Antioxidant Activity of Plant Extracts—ABTS Assay

The ABTS (2,2′-azinobis-(3-ethylbenzthiazolin-6-sulfonic acid)) assay measures the ability of an antioxidant to stabilize the ABTS radical cation (ABTS^•+^). The ABTS^•+^ is a green-blue chromophore produced through a reaction between 7 mM aqueous ABTS and 2.45 mM potassium persulfate (K_2_S_2_O_8_) mix in a ratio of 1:1 and incubated overnight (12–16 h) at room temperature in the dark. The ABTS solution was diluted to obtain 0.7 absorbance at 734 nm. In a 96-well microplate, a 10 μL sample of plant extract or Trolox as a positive control was mixed with 190 μL of ABTS radical solution and incubated for 30 min at room temperature in the dark. The blank well was obtained by mixing 10 μL of water and 190 μL of ABTS radical solution. For each test, three replicates were performed. The samples and blank absorbance were determined at 734 nm wavelengths in a microplate reader (Synergy H4, BioTek, Winooski, VT, USA; Agilent, Santa Clara, CA, USA). Radical scavenging activity (RSA) of plant extracts was calculated using the following formula:

% RSA = [(Abs _ABTS_ − Abs _Sample_)/Abs _ABTS_] × 100 (1)

where

% RSA—percent of radical scavenging activity
Abs _ABTS_—absorbance of ABTS
Abs _Sample_—absorbance of a sample

The antiradical activity of plant extracts was also expressed as EC50 (mg/mL), the concentration of the sample required to cause 50% ABTS inhibition. The EC50 value was calculated by a graphical method as the effective concentration resulting in 50% inhibition of radical formation. TEAC (Trolox equivalent antioxidant capacity) was calculated from the following Equation:

TEAC = EC50 _Trolox_/EC50 _Sample_ (2)

Trolox equivalent (TE) was calculated as mg TE on 1 g of dried plant extract. It is worth mentioning that the presented calculation does not take into account ABTS^•+^ radical cation self-bleaching. The ABTS self-bleaching calculations include the difference in the initial ABTS absorbance and the ABTS absorbance at the time of samples measure [57]. The decrease rate and absolute absorbance loss due to the self-bleaching of ABTS^•+^ radical-cations were maximal during the first 5–10 min, then declined steadily to reach generally negligible rates [58,59]. However, due to the technical aspect of the analysis, the absorbance were read no earlier than 15 min after adding ABTS^•+^ to analyzed samples when the impact of ABTS self-bleaching on the results obtained should not be so relevant.

### 4.6. Determination of Antioxidant Activity of Plant Extracts—DPPH Assay

In a 96-well microplate, samples of methanolic plant extracts or Trolox as a positive control were mixed with DPPH (2,2-diphenyl-1-picrylhydrazyl) radical solution at 0.2 mM in MeOH in a ratio of 1:1 and incubated for 30 min at room temperature in the dark. The blank well was obtained by mixing 100 μL of methanol and 100 μL of DPPH radical solution. For each test, three replicates were performed. The samples and blank absorbance were determined at 515 nm wavelengths in a microplate reader (Synergy H4, BioTek, Agilent, USA). Radical scavenging activity (RSA) of plant extracts was calculated using the following formula:

% RSA = [(Abs _DPPH_ − Abs _Sample_)/Abs _DPPH_] × 100 (3)

where

% RSA—percent of radical scavenging activity
Abs _DPPH_—absorbance of DPPH
Abs _Sample_—absorbance of a sample

The antiradical activity of plant extracts was also expressed as EC50 (mg/mL), the sample concentration required to cause 50% DPPH inhibition. The EC50 value was calculated by a graphical method as the effective concentration resulting in 50% inhibition of radical formation. TEAC was calculated from the following Equation:

TEAC = EC50 _Trolox_/EC50 _Sample_ (4)

Trolox equivalent (TE) was calculated as mg TE on 1 g of dried plant extract.

### 4.7. 3-(4,5-Dimethylthiazol-2-Yl)-2,5-Diphenyltetrazolium Bromide (MTT)—Based Viability Assay

The cytotoxicity of the plant extracts was examined by MTT assay (3-(4,5-dimethylthiazol-2-yl)-2,5-diphenyltetrazolium bromide). The HaCaT and Hs27 cells were seeded at a density of 8 × 10^4^ cells/mL in a 96-well micro-culture plate at 100 μL/well and incubated at 37 °C and 5% CO_2_ overnight. The following day, stock solutions of plant extracts were added in the final concentration range of 250, 125, 65.5, 31.25, and 15.62 μg/mL, whereas the control cells were treated with the equivalent volume of medium. Moreover, a background (plant extract with medium without HaCaT/Hs27 cells) was also performed. After 24 h, 48 h, and 72 h of incubation with the tested plant extracts, the MTT (1 mg/mL) was added, and the plates were incubated at 37 °C for 1 h. Subsequently, the MTT was removed, and 100 μL of dimethyl sulphoxide (DMSO) was added. The prepared plates were left on the plate shaker for 5 min. Optical densities were measured at 570 nm using a microplate reader (Synergy H4, BioTek, Agilent, USA). All measurements were carried out in a minimum of three replicates. The background was subtracted from the absorbance of cells treated with plant extracts to exclude plant extracts (compounds with redox properties) interfere with MTT.

Do not contain that may interfere with MTT.

### 4.8. Scratch Wound Healing Assay

The scratch wound healing assay was performed to determine the effects of plant extracts on the migration of HaCaT and Hs27 cells. The cells (7 × 10^5^ cells/mL) were dispersed in DMEM with 10% FBS and antibiotics (100 U/mL penicillin, 100 μg/mL streptomycin), and applied into each well of ibidi Culture-Insert 4 Well (Ibidi GmbH, Gräfelfing, Germany) placed in a 12-well plate within 24 h with 5% CO2 at 37 °C. When the cells formed a confluent monolayer, the inserts were removed, cellular debris was extracted, and washed with PBS. Pictures (4×) were taken after 0 h (initial time), 24 h, 48 h, and 72 h (HaCaT cells), and 4 h, 6 h, and 24 h (Hs27 cells) of incubation with plant extracts (25 and 50 μg/mL) in 1 ml of fresh DMEM medium without FBS and antibiotics. The wound closure area of the scratch was measured using ImageJ software (Version 1, National Institute of Health, Bethesda, MD, USA). The percentage of wound closure was calculated using the formula:

Wound closure (%) = (Initial scratch area − Final scratch area)/Initial scratch area) × 100(5)

### 4.9. Collagen Assay

Fibroblast Hs27 cells were seeded at an initial density of 8 × 10^4^ cells/mL in a 96-well plate in DMEM containing 10% FBS and antibiotics (100 U/mL penicillin, 100 μg/mL streptomycin), and then incubated at 37 °C and 5% CO_2_ overnight. After 24 h, plant extracts were added to the growth medium at a final concentration of 50 μg/mL and 25 μg/mL. Ascorbic acid at a concentration of 25 μg/mL was a positive control. Cells without plant extracts and ascorbic acid served as negative controls. After 48 h incubation at 37 °C with 5% CO_2_, the supernatants were collected. The amount of collagen was assayed using Collagen Assay Kit (Merck, Darmstadt, Germany), and measured by a microplate reader (Synergy H4, BioTek, Agilent, USA) at λ_ex_ = 375/λ_em_ = 465 nm. The amount of collagen was calculated based on a standard curve of collagen (0–50 μg/mL).

### 4.10. Statistical Analysis

Results are represented as mean ± s.e.m. of three independent experiments. Statistical analysis was performed using GraphPad Prism 5.0 software (GraphPad Software Inc., San Diego, CA, USA). Significance was determined using a one-way ANOVA analysis. The statistical significance of the differences was indicated in the figures with asterisks as follows: * *p* ≤ 0.05, ** *p* ≤ 0.01, and *** *p* ≤ 0.001.

## Figures and Tables

**Table 1:** Minimum inhibitory concentration (MIC), minimum bactericidal concentration (MBC), and minimum fungicidal concentration (MFC) values of various plant extracts against bacteria and yeast.

Plant Extracts	*P. aeruginosa*		*S. aureus*		*C. albicans*	
	MIC [mg/mL]	MBC [mg/mL]	MIC [mg/mL]	MBC [mg/mL]	MIC [mg/mL]	MFC [mg/mL]
*V. vinifera*	3.13	6.25	1.56	3.13	>50.00	>50.00
*C. cardunculus*	25.00	50.00	3.13	6.25	>50.00	>50.00
*P. sylvestris*	3.13	6.25	3.13	6.25	>50.00	>50.00
*S. aromaticum*	0.39	0.78	0.39	0.78	>50.00	>50.00
*R. rosea*	6.25	12.50	6.25	12.50	>50.00	>50.00
*S. baicalensis*	>50.00	>50.00	25.00	50.00	>50.00	>50.00
*S. officinalis*	>50.00	>50.00	3.13	6.25	>50.00	>50.00
*R. canina*	0.20	0.39	6.25	12.50	>50.00	>50.00
*B. serrata*	0.78	1.56	1.56	3.13	>50.00	>50.00
*P. incarnata*	25.00	50.00	6.25	12.50	>50.00	>50.00
Novobiocin	<0.01	<0.01	0.31	0.66	-	-
Cycloheximide	-	-	-	-	<0.01	<0.01

**Table 2:** ABTS and DPPH radical scavenging activities (EC50 values), Trolox equivalent antioxidant capacities (TEAC) and Trolox equivalent (TE) of plant extracts.

Plant Extract	ABTS			DPPH		
	EC50 [mg/mL]	TEAC	mg TE/g	EC50 [mg/mL]	TEAC	mg TE/g
*V. vinifera*	0.078	2.216	2437	0.005	1.234	1538
*C. cardunculus*	2.275	0.076	78.62	0.300	0.019	48.55
*P. sylvestris*	0.309	0.559	535.4	0.008	0.739	848.5
*S. aromaticum*	0.264	0.654	874.8	0.015	0.383	434.8
*R. rosea*	1.194	0.145	164.8	0.038	0.152	140.0
*S. baicalensis*	19.602	0.009	7.895	0.016	0.353	36.68
*S. officinalis*	2.909	0.059	54.31	1.949	0.003	19.52
*R. canina*	0.121	1.425	1362	0.006	1.018	1052
*B. serrata*	15.700	0.011	10.68	6.092	0.001	0.965
*P. incarnata*	2.036	0.085	86.18	0.472	0.012	14.93
*Trolox*	0.173	1.000	-	0.006	1.000	-

## Data Availability

Data are contained within the article.

## Supplementary Materials

The following supporting information can be downloaded at https://www.mdpi.com/article/10.3390/ijms26157490/s1.
