# Supplementary material for: Plant Extracts as Modulators of the Wound Healing Process—Preliminary Study
Source: Int J Mol Sci. 2025 Aug 2;26(15):7490. doi: 10.3390/ijms26157490 (PMC12347978; doi:10.3390/ijms26157490)
Supplement: Supplementary file 1 [file ijms-26-07490-s001.zip › Herman Anna - Figure S2.pdf]

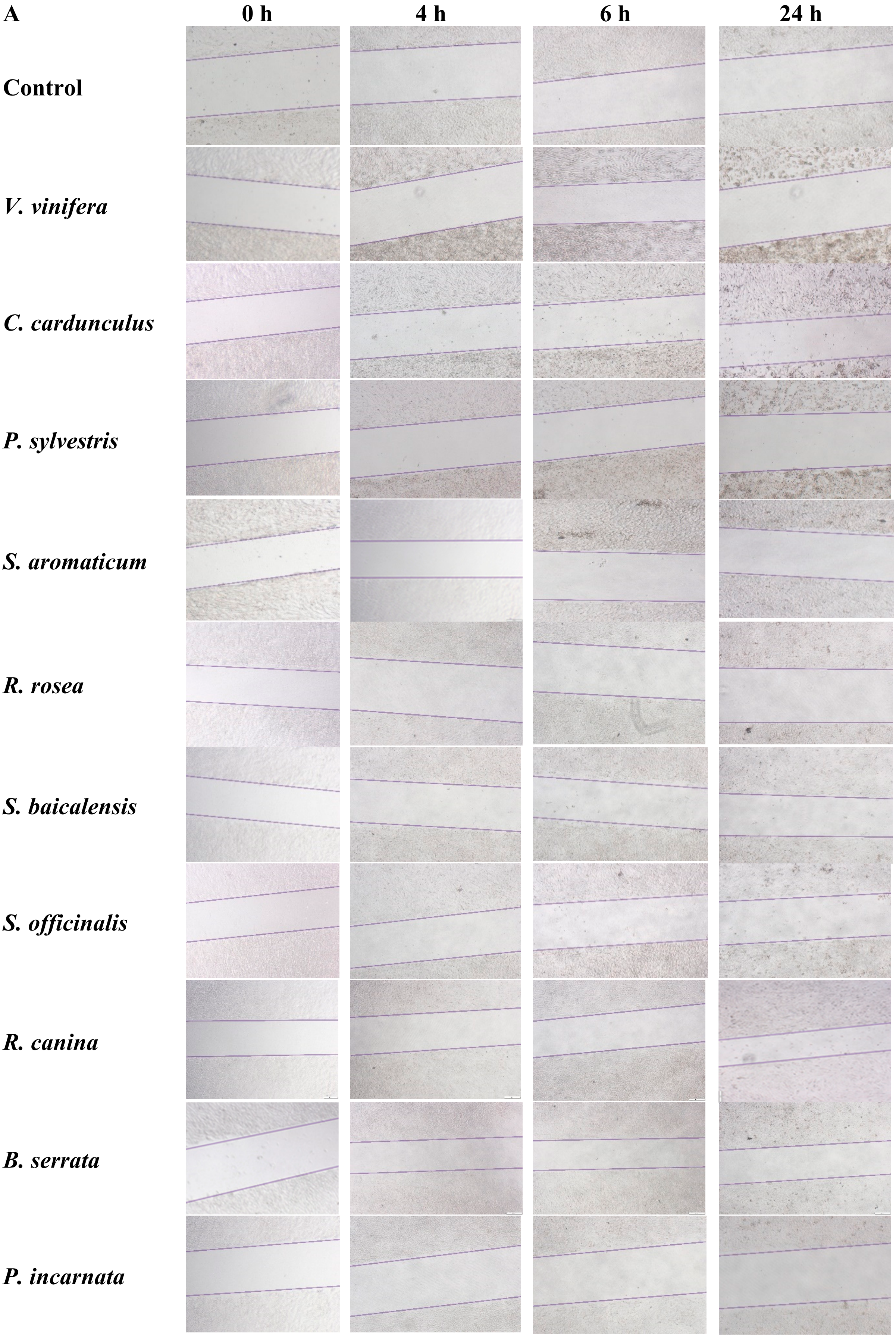
A
0 h
4 h
6 h
24 h
Control
V. vinifera
C. cardunculus
P. sylvestris
S. aromaticum
R. rosea
S. baicalensis
S. officinalis
R. canina
B. serrata
P. incarnata

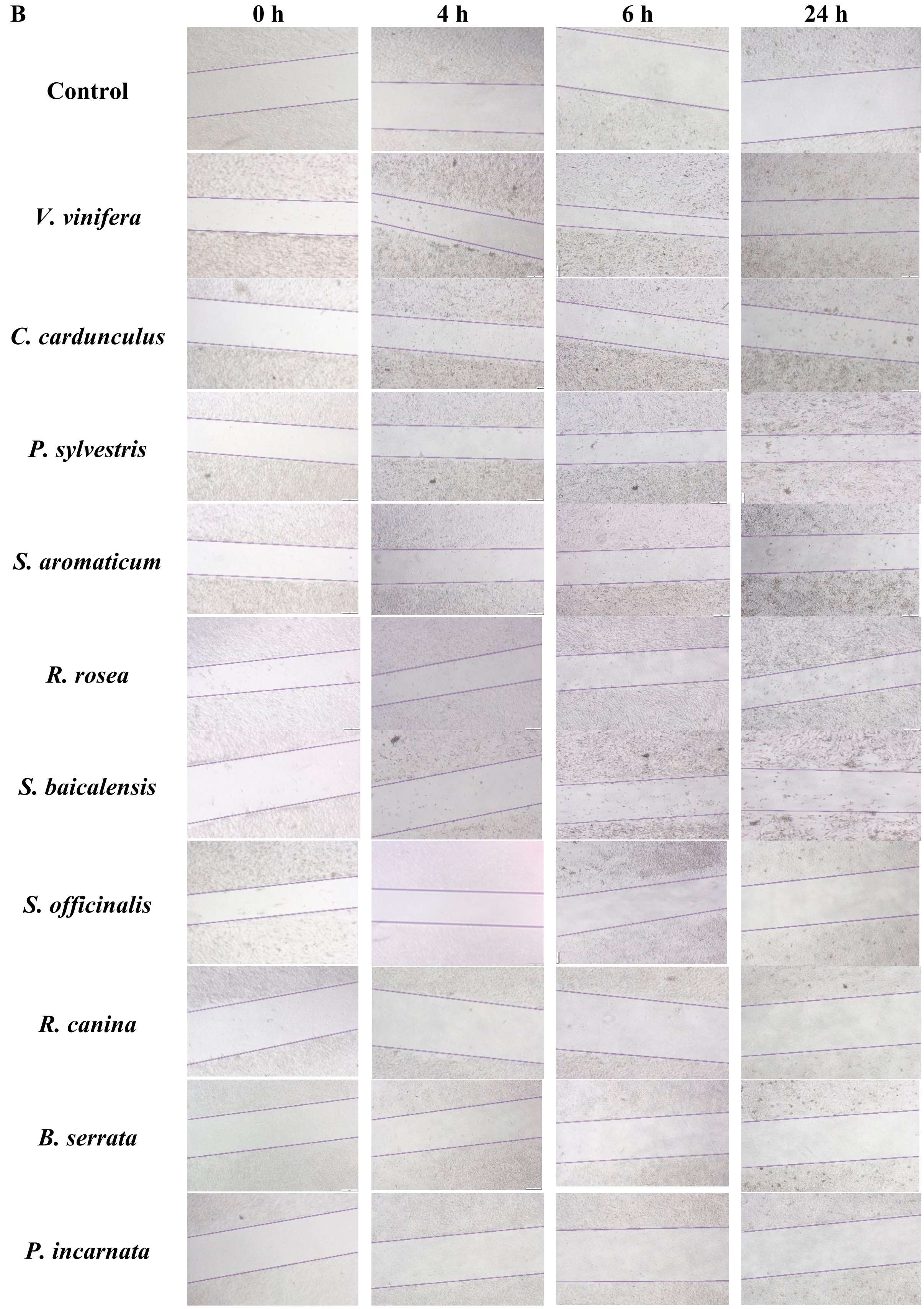

Figure S2. The microscopic images of the untreated human fibroblasts (Hs27) and treated with 50 µg/mL (**A**) and 25 µg/mL (**B**) plant extracts after 0h, 4h, 6h, and 24h incubation. All images were recorded at 4× magnification, scale bar: 200 µm.
